# Is Chlorate of Potassium in Ordinary Doses Ever Poisonous?

**Published:** 1883-03

**Authors:** E. Ingals


### ARTICLE IV.

### IS CHLORATE OF POTASSIUM IN ORDINARY DOSES EVER POISONOUS? By E. INGALS, M. D.

In an editorial in the *Medical Record*, of January 13, on "Directions Regarding the Administration of Medicines," we are told that a girl fourteen years old, suffering from follicular inflammation of the fauces, had a mixture prescribed by her physician, of which she was to take a teaspoonful three times daily. In addition, the physician told her to dissolve two teaspoonfuls of chlorate of potassium in a tumbler of water, and with this to gargle the throat at intervals. The mother, misinterpreting the directions, gave the potash internally in doses of two teaspoonfuls of the saturated solution four times daily, with an equal quantity of the "mixture." This would give about eight grains of the chlorate to a dose, or thirty-two grains in twenty-four hours. Symptoms of poisoning, of which the child died, were developed on the second day. The statement leaves the impression that poisoning was attributed to the chlorate of potassium. It would be interesting to know of what the "mixture" that was administered along with it was composed. I am sure that less than ten grains of chlorate of potassium administered four times daily to a girl of fourteen years, so far from being poisonous, would hardly produce any appreciable effects. This, however, does not militate against the proposition that the physician should be precise and clear in his directions to both nurse and patient.

